# Feasibility and outcomes of ERAS protocol in elective cT4 colorectal cancer patients: results from a single-center retrospective cohort study

**DOI:** 10.1186/s12957-021-02282-7

**Published:** 2021-07-02

**Authors:** Vittoria Bellato, Yongbo An, Daniele Cerbo, Michela Campanelli, Marzia Franceschilli, Krishn Khanna, Bruno Sensi, Leandro Siragusa, Piero Rossi, Giuseppe S. Sica

**Affiliations:** 1 Minimally Invasive Unit, Department of Surgery, Università “Tor Vergata”, Viale Oxford 81, 00133 Rome, Italy; 2 Department of Colorectal Surgery, St Mark’s Academic Hospital, London, UK; 3 Department of General Surgery, Beijing Friendship Hospital, Capital Medical University, Beijing, China; 4 Department of Surgery, Rush University Medical Center, Chicago, IL USA

**Keywords:** Colorectal cancer, ERAS, Fast track, T4, Enhanced recovery

## Abstract

**Background:**

Programs of Enhanced Recovery After Surgery reduces morbidity and shorten recovery in patients undergoing colorectal resections for cancer. Patients presenting with more advanced disease such as T4 cancers are frequently excluded from undergoing ERAS programs due to the difficulty in applying established protocols. The primary aim of this investigation was to evaluate the possibility of applying a validated ERAS protocol in patients undergoing colorectal resection for T4 colon and rectal cancer and to evaluate the short-term outcome.

**Methods:**

Single-center, retrospective cohort study. All patients with a clinical diagnosis of stage T4 colorectal cancer undergoing surgery between November 2016 and January 2020 were treated following the institutional fast track protocol without exclusion. Short-term postoperative outcomes were compared to those of a control group treated with conventional care and that underwent surgical resection for T4 colorectal cancer at the same institution from January 2010 to October 2016. Data from both groups were collected retrospectively from a prospectively maintained database.

**Results:**

Eighty-two patients were diagnosed with T4 cancer, 49 patients were included in the ERAS cohort and 33 in the historical conventional care cohort. Both, the mean time of tolerance to solid food diet and postoperative length of stay were significantly shorter in the ERAS group than in the control group (3.14 ± 1.76 vs 4.8 ± 1.52; *p* < 0.0001 and 6.93 ± 3.76 vs 9.50 ± 4.83; *p* = 0.0084 respectively). No differences in perioperative complications were observed.

**Conclusions:**

Results from this cohort study from a single-center registry support the thesis that the adoption of the ERAS protocol is effective and applicable in patients with colorectal cancer clinically staged T4, reducing significantly their length of stay and time of tolerance to solid food diet, without affecting surgical postoperative outcomes.

## Background

Enhanced Recovery After Surgery (ERAS) was introduced in the late 1990s and consisted of a series of evidence-based guidelines covering the entire perioperative period. ERAS is applied by a multidisciplinary team in a hospital setting, with the aim of reducing surgical stress and maintaining postoperative physiological functions [1]. This approach has been shown to reduce morbidity, improve recovery, and shorten length of stay (LOS) after gastrointestinal surgery and specifically in colorectal patients [2–6]. Updates and changes in the guidelines are provided by the ERAS Society (www.erassociety.org); the latest consensus for colorectal surgery was published by Gustafsson et al. in 2018 [7].

Even though the benefits of an enhance recovery approach can be successfully applied in most instances to colon and rectal cancer surgery, patients diagnosed with T4 colorectal cancer represent a specific subgroup, frequently composed of fragile patients, whose advanced disease may require multiorgan resections and open surgery. This has historically made T4 colorectal cancer patients less suitable for ERAS protocols, due to an expected higher rate of intra and postoperative complications and reduced compliance, of both patients and caregivers. These characteristics, combined with the inapplicability of some of the ERAS principals such as no use of abdominal drains, early removal of urinary catheters, early feeding, and mobilization, are usually considered to be jeopardizing one of the principal outcomes of ERAS that is prompt discharge.

The impact of enhanced recovery programs on postoperative outcomes in this subset of patients has never been addressed in literature. The majority of studies on the topic either excluded T4 patients due to higher rates of complications or adopted a homogeneous patient sampling analyzing all stages together, with cT4 stages generally account for less than 15% of colorectal malignancies at diagnosis [8–12].

The aim of the study was to investigate the feasibility of an established ERAS protocol in patients with a diagnosis of T4 colorectal cancer and to compare in-hospital outcomes of patients who underwent the ERAS protocol with historic controls.

## Materials and methods

### Study design and population

Two groups of patients were included and analyzed.

- *Group A*. Patients undergoing surgical resection with a clinical diagnosis of stage T4 colorectal cancer (defined as 8th Union of International Cancer Control (UICC) TNM classification of Malignant Tumor) between November 2016 to January 2020 at Minimally Invasive Surgery Unit of Tor Vergata University Hospital treated according to our newly established ERAS protocol, were included in the study group.
- *Group B*. Patients that had colorectal resections for T4 cancers and that underwent standard perioperative care in the same academic tertiary care institution from January 2010 to October 2016 were included in the historical control group.

All patients > 18 years old with diagnosis of T4 adenocarcinoma of the colon or rectum were included.

Exclusion criteria were the presence of synchronous cancers, failure to perform colonic or rectal resection and emergency surgery setting.

The decision to exclude patients who underwent resection in emergency setting was made based on inapplicability of preoperative ERAS items and lack of patients counseling.

Data of both cohorts were collected retrospectively from a prospectively maintained database. Feasibility of the ERAS protocol in the group A was recorded for each of the adopted items. ERAS items are compared to the perioperative measures adopted in the conventional care group in Table 1. ERAS items were those of the guidelines available in 2016, published in 2012 by Gustafsson et al.

**Table 1:** ERAS items compared to the perioperative measures adopted in the conventional care

ERAS items adopted	Conventional care
**Preadmission information, education, and counseling (dedicated preoperative counseling about ERAS protocol)**	**Not applicable**
**Preoperative optimization (increasing preoperative exercise and avoid smoking and drinking alcohol 1-month prior surgery)**	**None**
**Preoperative fasting and carbohydrate loading (fluid up to 2 h and solid up to 6 h prior of induction of anesthesia, preoperative carbohydrate loading)**	**Preoperative fasting 12 h prior of induction of anesthesia**
**Preoperative thromboprophylaxis with LMWH; extended prophylaxis for 28 days for colorectal cancer patients**	**Same protocol**
**Avoid pre-anesthetic medication (midazolam) or, if necessary, administer short-acting intravenous drugs**	**Pre-anesthetic medication routinely used**
**Antimicrobial prophylaxis (preoperative intravenous antibiotics) and skin preparation**	**Antibiotic prophylaxis prolonged for 48 h postoperatively**
**Multimodal approach to PONV preoperatively (minimal preoperative fasting, carbohydrate loading), intraoperative (anesthetic PONV prevention), and postoperatively (add antiemetic that were not used for prophylaxis)**	**Treating PONV with antiemetics only once has already appeared**
**Standard anesthetic protocol allowing rapid awakening**	**None**
**Perioperative fluid management (balanced crystalloid, intraoperative fluids administration guided by flow measurements in open surgery, vasopressor in management of epidural-induced hypotension, early enteral administration of fluids)**	**Balanced crystalloid**
**Preventing intraoperative hypothermia (intraoperative maintenance of normothermia with warming device and warmed intravenous fluid to keep temperature > 36 °C)**	**Use of warmed intravenous fluid**
**Minimally invasive surgical access recommended (7/9 surgeon MIS trained)**	**Few surgeons (2/9) MIS trained**
**Drainage of the peritoneal cavity and pelvis (routine drainage discouraged, early removal in POD 1/2 when no blood or purulent output)**	**Abdominal drainage routinely used, removed when output < 100 ml**
**Nasogastric Intubation not inserted, unless gastric distension in presence of bowel occlusion**	**Nasogastric tube routinely used and removed when output < 200 ml/day**
**Postoperative analgesia (FANS +/- Tap block)**	**PCA with morfine**
**Urinary drainage (not routinely used, removed when possible after postoperative day 1/2) T4 patients with pelvic mass or with bladder involvement are considered high risk for urinary retention, therefore maintained urinary catheter at least until day 3**	**Urinary catheter removal after postoperative day 4/5 or when patients were able to mobilize**
**Postoperative glycemic control (use of stress reducing element of ERAS to minimize hyperglycemia, insulin treatment in ICU and ward setting when required)**	**Standard glycemic control**
**Postoperative nutritional care (postoperative early enteral feeding and nutritional screening)**	**Enteral feeding resumed when bowel movement present and nasogastric tube output < 100 ml**
**Early mobilization within first postoperative day. Patient’s counseling, dedicated nursing staff, and physiotherapists to early mobilization**	**Mobilization after removal of Foley catheter. Never before postoperative day 3**

Since the indication on bowel preparation has undergone several changes during the study period that was analyzed, this ERAS item was not included in the analysis.

The study was conducted in accordance with STROBE criteria (http://strobe-statement.org/) and registered under ClinicalTrials.gov, NCT04466696.

### Endpoints

The primary endpoint was postoperative length of stay (LOS), defined as the number of postoperative days (POD) of in-hospital recovery. Secondary endpoints were prolonged LOS (PLOS), defined as discharge after the eighth POD, time to postoperative solid oral intake, defined as tolerance of solid diet and the time of first bowel movement and first flatus. Other endpoints were overall morbidity (according to the Clavien-Dindo Classification), reoperation rates, and 30-day readmission and mortality rates. Compliance to the single items of the protocol in the ERAS group was also analyzed.

### Statistical analysis

Descriptive statistical methods were used to characterize the sample. Data are presented as median, range, and standard deviation (DS). We used the chi-squared test to compare discrete variables. An independent sample t-test was used for continuous, normally distributed data. A p value of < .05 was considered statistically significant. Statistical analyses were performed using SPSS (version 23, IBM Corp, Armonk, NY).

### Compliance with ethical standards

Informed consent was obtained from all individual participants included in the prospective portion of the study. The ethical committee of our institution (CEI-TV) under protocol no. 410/20 gave approval of use of patients’ information for this study.

## Results

### Study population

From January 2010 to January 2020, 595 patients diagnosed with colorectal cancer underwent surgical resection at Minimally Invasive Unit of Tor Vergata Hospital, of which 105 were diagnosis with cT4 colorectal cancer. Eighty-two cT4 patients treated electively are included in the study analysis, while 23 cT4 patients that were operated on in emergency are excluded. Thirty-three cT4 elective patients that were operated on between January 2010 and October 2016 were treated with conventional care, thus included in the control group, while 49 patients operated on between November 2016 and January 2020 were treated with ERAS protocol and included in the ERAS group.

Patient’s demographics are summarized in Table 2. The two groups were comparable with respect to age, gender, BMI, ASA score, comorbidities (including diabetes, hypertension, heart and respiratory diseases), surgical approach, surgical procedures, and need of multi-visceral resection.

**Table 2:** Patients demographics

Parameters	Group A (ERAS T4) (***n*** = 49)	Group B (standard care) (***n*** = 33)	P
**Age (mean, SD)**	**69.02 ± 12.8**	**66.51 ± 10.12**	**0.379**
**Sex**			
**Male**	**28 (57.1%)**	**18 (54.5%)**	**1**
**Female**	**21 (42.9%)**	**15 (45.5%)**	
**Preoperative BMI (mean, SD)**	**25.86 ± 4.15**	**24.8 ± 3.77**	**0.243**
**ASA score**			
**1**	**5 (10.2%)**	**1 (3%)**	**0.399**
**2**	**25 (51%)**	**17 (51.5 %)**	
**3**	**18 (36.7%)**	**13 (39.5%)**	
**4**	**1 (2.1%)**	**2 (6%)**	
**Comorbidity**			
**Diabetes**	**7 (14.3%)**	**3 (9.1%)**	**0.735**
**Hypertension**	**24 (49%)**	**22 (66.6%)**	**0.459**
**Heart disease**	**13 (26.5%)**	**5 (15.1%)**	**0.425**
**Respiratory disease**	**6 (12.2%)**	**5 (15.1%)**	**0.754**
**Preoperative albumin (gr/dl) (mean, SD)**	**3.77 ± 0.75**	**3.31 ± 0.65**	**0.005**
**T4**			
**A**	**34 (69.3%)**	**26 (78.8%)**	**0.733**
**B**	**15 (31.7%)**	**7 (21.2%)**	
**Surgical approach**			
**Open**	**26 (53.1%)**	**19 (57.6%)**	**0.852**
**Laparoscopy**	**18 (36.7%)**	**10 (31.3%)**	**0.823**
**Converted**	**5 (10.2%)**	**4 (12.1%)**	
**Surgical procedure**			
**Right hemicolectomy**	**11 (22.4%)**	**17 (51.5%)**	**0.079**
**Left hemicolectomy**	**16 (32.6%)**	**5 (15.2%)**	
**Anterior rectal resection**	**18 (36.8%)**	**9 (27.3%)**	
**Other (1 subtotal colectomy, 1 miles, 1 Hartmann, 1 proctocolectomy)**	**4 (8.2%)**	**2 (6 %)**	
**Multi-visceral resection**			
**Yes**	**15 (31.7%)**	**9 (27.3%)**	**1**
**No**	**34 (69.3%)**	**24 (72.7%)**	

### ERAS compliance in group A

ERAS protocol compliance to each item is shown in Table 3. For half of the items, the overall compliance was 80%. For the rest of the items, the level of compliance was lower: pre-anesthetic medication with midazolam (67%), intraoperative fluid management (69%), minimally invasive surgical approach (T4a = 50%, T4b = 7%), no use of peritoneal drainage (10%), postoperative analgesia (47%), early urinary catheter removal (55%), postoperative nutritional care (69%), and early mobilization (61%).

**Table 3:** ERAS protocol compliance

ERAS item		Compliance (%)
**Preadmission information, education and counseling**		**100**
**Preoperative optimization**		**100**
**Preoperative fasting and carbohydrate loading**		**100**
**Thromboprophylaxis**		**100**
**Pre-anesthetic medication**		**67**
**Antimicrobial prophylaxis and skin preparation**		**100**
**Prevention of nausea and vomiting (PONV)**		**100**
**Standard anesthetic protocol**		**82**
**Perioperative fluid and electrolyte therapy**		**69**
**Preventing intraoperative hypothermia**		**100**
**Minimally invasive surgical access**	**T4a**	**50**
	**T4b**	**7**
**Drainage of the peritoneal cavity and pelvis**		**10**
**Nasogastric intubation**		**80**
**Postoperative analgesia**		**47**
**Urinary drainage**		**55**
**Postoperative glycemic control**		**100**
**Postoperative nutritional care**		**69**
**Mobilization**		**61**

### Postoperative outcomes

Postoperative outcomes of the two groups are displayed in Table 4. Concerning the primary outcome, LOS was shown to be significantly lower in the ERAS cohort, with a mean of 6.93 ± 3.76 in the ERAS group compared to 9.50 ± 4.83 in the conventional care cohort (*p* = 0.0084). As secondary outcomes, mean time to postoperative solid oral intake tolerance was significantly shorter in the ERAS cohort compared with the conventional cohort (3.14 ± 1.76 vs 4.8 ± 1.52; *p* < 0.0001). The remaining recorded postoperative outcomes did not show any significant differences between the two cohorts; complications rates were similar.

**Table 4:** Postoperative outcomes

*Parameters*	*Group A (ERAS T4) (n = 49)*	*Group B (standard care) (n = 33)*	*P*
First flatus (mean, SD)	2.46 ± 1.06	3.06 ± 1.31	**0.025**
First bowel movement (mean, SD)	3.83 ± 1.83	4.4 ± 1.56	0.1467
Mean time of tolerated food intake, days (mean, SD)	3.14 ± 1.76	4.8 ± 1.52	**0.0001**
Postoperative length hospital stay, days (mean, SD)	6.93 ± 3.76	9.50 ± 4.83	**0.008**
Prolonged length of stay (days)	14 (29.8%)	16 (48.5%)	0.283
Complications (No.)	13 (26.5%)	6 (18.2%)	0.604
Anastomotic leak	3 (6.1%)	2 (6.1%)	1.
SSI	2 (4.1%)	4 (12.1%)	0.234
Pneumonia	2 (4.1%)	3 (9.1%)	0.645
Postoperative blood transfusion	11 (22.4%)	4 (12.1%)	0.395
Clavien-Dindo			
0	35 (71.5%)	27 (81.8%)	0.159
1	1 (2%)	0	
2	9 (18.3%)	3 (9.1%)	
3a	0	0	
3b	4 (8.2%)	0	
4	0	2 (6.1%)	
5	0	1 (3%)	
Reoperation rate	2 (4.1%)	2 (6.1%)	1.
30 days readmission rate	2 (4.1%)	1 (3%)	1.
30 days mortality	0 (0%)	1 (3%)	0.410

## Discussions

The results of this study support the thesis that the adoption of the ERAS protocol is effective and applicable in patients with colorectal cancer clinically staged T4, reducing significantly their LOS without affecting surgical postoperative outcomes. It is interesting to note that the decreased compliance with postoperative items does not affect the main ERAS outcome, suggesting the importance of the pre/intraoperative phase, especially patient counseling regarding advantages and duties of the ERAS protocol.

Since the first report by Kehlet et al. [13] back in 1997, the adoption of fast-track protocols in elective colorectal surgery has been shown to reduce postoperative length of stay and results in a faster recovery when compared to traditional care [14–16].

However, the impact of fast-track protocols on patients affected by advanced colorectal cancer has never been investigated. Many studies that compared ERAS to standard of care analyze all stages of colorectal cancer as a single group [17, 18]. As a matter of fact, T4 colorectal cancer patients undergoing surgical resections are a minority (5–8%) [19] and probably their weight is not influential. On the contrary, in other trials, patients with complex and/or multiorgan resections and/or patients who were previously treated with neoadjuvant chemo radiotherapy are excluded in order to limit the heterogeneity of the study population [20–22]. The primary reasons being that a larger number of patients are exposed to more complex surgery and, therefore, are more likely to develop postoperative complications. Clearly, they would represent a mark in the analysis of outcomes such as complication rates and LOS.

Nevertheless, patients treated with ERAS protocol from Gatt, Fosmo, and Feng, who all excluded either multi-visceral resections or advanced stage of disease from their studies, do not differ from those of Gouvas and Nanavati who included T4 resections, with regard to median postoperative LOS (5 days; 5 days and 5.5 days vs 5.5 days and 4.75 days, respectively).

Another explanation for the exclusion of advanced stage colorectal cancer patients in ERAS trials, lies in the belief that they would have a more difficult compliance with some specific perioperative items such as the avoidance of abdominal drains, early urinary catheter removal, early feeding, and mobilization.

Lately, the nature of these items has been the focus of debate, since they could be considered markers of both protocol compliance and recovery [23]. Since many postoperative items are strongly linked to the onset of postoperative complications, it is difficult to figure out whether a given patient had better recovery because he was eating and ambulating early or whether he tolerated early eating and walked early thanks to rapid recovery. For the aforementioned reasons, we have included in our study group all cT4 patients undergoing colorectal resections under an established ERAS protocol, with no exception. All patients were included in the analysis of results even in case of complex surgery and multiorgan resections. However, in such cases, a part of the ERAS principles could not be respected and the overall postoperative compliance was scarce. Despite a lower compliance, the study group obtained better outcomes in terms of PLOS and time to tolerate solid food compared to patients treated with traditional care.

Some other institutions focused their research on special subgroups of patients undergoing colorectal surgery such as patients affected by Crohn’s disease [24–33] and elderly patients [34–39], proving that even fragile patients could benefit from a faster recovery obtained through the ERAS protocol. Small series by Feroci et al. [40] and Kisialeuski et al. [41] confirmed a lower adherence to overall ERAS items in elderly compared to younger patients, especially regarding mobilization and intravenous fluid therapy duration, while they recorded similar median postoperative LOS in the ERAS groups regardless of compliance.

In our study, we found a high level of compliance with regard to the application of preoperative ERAS items. This is probably due to the extensive effort made to provide counseling the patients and to the fact that patients operated in emergency were excluded.

However, a lower level of compliance for both caregivers and patients, was found for some intra and postoperative items, inter alia minimally invasive approach (50% for T4a VS 7% for T4b), drainage of abdominal cavity (10%), postoperative analgesia (47%), and early urinary catheter removal (55%), which can to be related to the advanced nature of the disease.

A minimal invasive approach is considered a fundamental tool to maximize the results of enhanced recovery and two RCTs already compared the adoption of ERAS protocols between open and laparoscopic surgery with or without an implementation with fast track. A combination of ERAS with laparoscopy results in a significant faster recovery compared with all other combinations (open + ERAS, laparoscopy + standard, open + standard). However, the open approach + ERAS also reduces the LOS, thus demonstrating a success of the ERAS program [42, 43].

Even if not recommended by the guidelines, feasibility and safety of laparoscopic resection in T4 colorectal tumors have been investigated by some series concluding that, despite the increased odds of conversion, in specialized centers and selected patients, laparoscopy can be applied to patients with T4 colorectal tumors without worsening long-term outcomes [44–54]. In our study, we treated laparoscopically with an R0 resection 37 patients out of 82; in nine patients, it was necessary to complete the operation with a conversion to open surgery. However, the number of patients who underwent laparoscopic resections did not differ significantly in the two groups (*p* = 0.83); therefore, the beneficial effects of laparoscopic surgery were present in equal manners in both the study group and the control group.

Although there are guidelines for implementing an enhanced recovery protocol for colorectal surgery, variation in the number and definition of protocol components, as well as variation in the criteria for adherence, contributes to difficulties in determining which components are most important for improving patient outcomes [55–58]. Our results suggest that a complex surgery with an expected overall lower compliance to ERAS items should not be a deterrent to fast-track application, considering the benefits in terms of faster recovery, with no differences in terms of complications.

Furthermore, we would also highlight a short LOS as crucial during the current COVID-19 pandemic, both in terms of resources optimization, then in terms of reduced risk of hospital acquired disease. The relevance of these considerations is amplified by the recent evidence of an overall increase number of advanced cancer patient’s receiving surgical treatment, related to the delay in diagnosis and treatment since the beginning of the pandemic [59–62].

Limitations of this analysis are the retrospective nature of the study, which is also a single-unit study, the lack of contemporaneous controls, and the small sample size of the study group. The small sample size does not permit a risk adjustment analysis for factors as complications and confounding factors; in the face of a huge average effect on the primary outcome, the lack of specificity of the effect is a major limitation. However, single-center trials provide the flexibility of approach necessary for clinicians and scientists to develop new treatments and can provide an important source for new therapeutic ideas. In fact, some ERAS strategies, such as antimicrobial prophylaxis, prevention of hypothermia, and thoracic epidural anesthesia during open surgery, but also laparoscopy in colon surgery, are considered the current “standard of care”; therefore, it may be considered unethical and difficult to perform randomized trials to evaluate the benefits of each of the ERAS items. Further multicentric prospective studies with lager sample size are warranted to help define the benefits of ERAS protocol in advanced colorectal patients.

## Conclusion

To our knowledge, this is the first study analyzing the feasibility of ERAS program exclusively in T4 cancer patients’ undergoing colorectal resections. These preliminary results, from a single unit, show that ERAS is feasible in T4 colorectal cancer patients and can enhance postoperative recovery.

## Data Availability

Data supporting reported results can be found in the database of Policlinico Tor Vergata (www.ptvonline.it). Data are protected and access availability must be obtained.
